# Temperature-Dependent Magnetic Response of Antiferromagnetic Doping in Cobalt Ferrite Nanostructures

**DOI:** 10.3390/nano6040073

**Published:** 2016-04-18

**Authors:** Adeela Nairan, Maaz Khan, Usman Khan, Munawar Iqbal, Saira Riaz, Shahzad Naseem

**Affiliations:** 1 Centre for High Energy Physics, University of the Punjab, Lahore 54000, Pakistan; muniqbal@yahoo.com; 2 Nanomaterials Research group, Physics Division, Pakistan Institute of Nuclear Science and Technology, Nilore, Islamabad 45650, Pakistan; maaz@impcas.ac.cn; 3 Institute of Physics, Chinese Academy of Sciences, Beijing 100190, China; usman@iphy.ac.cn; 4 Centre for excellence in Solid State Physics, University of the Punjab, Lahore 54000, Pakistan; saira_cssp@yahoo.com (S.R.); shahzad_naseem@yahoo.com (S.N.)

**Keywords:** co-precipitation, ferrites, zero field cooled (ZFC) and field cooled (FC) curves, magnetic anisotropy

## Abstract

In this work Mn*_x_*Co_1−*x*_Fe_2_O_4_ nanoparticles (NPs) were synthesized using a chemical co-precipitation method. Phase purity and structural analyses of synthesized NPs were performed by X-ray diffractometer (XRD). Transmission electron microscopy (TEM) reveals the presence of highly crystalline and narrowly-dispersed NPs with average diameter of 14 nm. The Fourier transform infrared (FTIR) spectrum was measured in the range of 400–4000 cm^−1^ which confirmed the formation of vibrational frequency bands associated with the entire spinel structure. Temperature-dependent magnetic properties in anti-ferromagnet (AFM) and ferromagnet (FM) structure were investigated with the aid of a physical property measurement system (PPMS). It was observed that magnetic interactions between the AFM (Mn) and FM (CoFe_2_O_4_) material arise below the Neel temperature of the dopant. Furthermore, hysteresis response was clearly pronounced for the enhancement in magnetic parameters by varying temperature towards absolute zero. It is shown that magnetic properties have been tuned as a function of temperature and an externally-applied field.

## 1. Introduction

Magnetic nanomaterials have been given special attention by the scientists due to their countless applications in the recent era of science. However, highly magnetic storage devices, sensors, ferrofluids, magnetic resonance imaging (MRI), transformers, refrigeration system, and several other technological pillars are highly dependent on these materials [1,2,3,4]. Magnetic spinel ferrites, on the other hand, have played an important role in these applications because of their thermal and chemical stabilities towards their overall magnetic response. The general representation of ferrites is given by MFe_2_O_4_, where M stands for metal ion from the 3*d* transition elements. The unit cell of spinel ferrites consists of 32 oxygen atoms with two lattice sites available for cation distribution *i.e.*, tetrahedral (A) and octahedral (B) lattice sites [5]. Theoretically, divalent ions occupy A sites and trivalent ions occupy B lattice sites in normal spinel structures. However, in the case of an inverse spinel structure half of the trivalent cations (*i.e.*, Fe^3+^) replace divalent ions at tetrahedral sites and the other half replace octahedral sites [6].

Among spinel ferrites cobalt ferrite (CoFe_2_O_4_) has long been the subject of study due to its unique properties, such as high coercivity, large magneto-crystalline anisotropy, moderate saturation magnetization, mechanical stability, large magnetostrictive coefficient, and large mechanical hardness [7]. Cobalt ferrite belongs to the inverse spinel group and it is one of the strongest candidates for various applications like magneto-optic recording material, microwave industries, drug delivery, gas sensors, and solar cells [8,9,10,11]. Control of size, morphology, and chemical composition of ferrite NPs allows tailoring its various properties for specific applications. Moreover, substitution of ferrite NPs with different elements can possibly enhance the magnetic characteristics of these materials. Manganese (Mn) ions can have high magnetic properties because of their large magnetic moments (5 µ_B_) per Mn^2+^ ion. Previous literature shows that magnetic properties of CoFe_2_O_4_ can be enhanced by substitution of Mn ions [12]. Additionally, due to possessing insulating properties, high T_C_ and uniaxial magnetic anisotropy, these ferrites are considered as good candidate for highly spin-polarized currents, magnetic tunnel junctions (MTJ’s) [13], and spin filtering devices, which consists of a ferromagnetic insulator layer sandwiched between a non-magnetic metallic (NMM) layer and a ferromagnetic metallic (FMM) layer [14]. Several methods have been employed for synthesis of NPs such as sol gel [15], ball milling [16], hydrothermal [17], co-precipitation [18], thermal plasma methods [19], and auto combustion [20]. Among these techniques co-precipitation is an inexpensive, simple, and low-temperature synthesis route of ferrite NPs.

In the present work, we synthesized magnetic NPs by substituting 20% Co with Mn ions in CoFe_2_O_4_ using the co-precipitation route. Our earlier studies have already been emphasized on Mn-doped CoFe_2_O_4_ NPs to investigate the room-temperature magnetic response [21]. Recently, we determined the structural- and temperature-dependent magnetic properties of Co_0.8_Mn_0.2_Fe_2_O_4_ NPs. Magnetic measurements have been performed at low temperatures from 5 K to 400 K to monitor the variation in magnetic properties of NPs.

## 2. Experimental Section

The NPs were synthesized by using a co-precipitation method. The stoichiometric amounts of 0.2 M cobalt chloride (CoCl_2_·6H_2_O), manganese chloride (MnCl_2_·4H_2_O), and 0.4 M iron chloride (FeCl_3_) were dissolved in distilled water taking 25 mL solution volume for each metal. After combining these solutions, a specific amount of oleic acid (50 µL) was added as a surfactant. 3 M (25 mL) solution of sodium hydroxide (NaOH) was slowly added to the mixture solution until a pH value of 12 was achieved. Reactants were continuously stirred during the process and then the mixture was heated at 80 °C for one hour. At this temperature the co-precipitation reaction takes place and desired ferrite NPs are formed [22]. After completing the reaction, the solution was allowed to cool down to room temperature. The obtained product was washed several times with deionized water and ethanol to remove the organic and inorganic impurities, if present, in the mixture. The solution was then centrifuged for 20 min at 3000 rpm and dried overnight at 100 °C. The dried sample was then ground into a fine powder and annealed at 600 °C for 6 h to obtain pure ferrite NPs. Afterwards, the sample was given a name Co_0.8_Mn_0.2_Fe_2_O_4_ (CMF).

Structural properties of CMF were carried out by recording the X-ray diffraction pattern for 2θ in the range 15°–80° (XRD: RIGAKU-D/MAX-2400, Beijing, China, Cu Kα, λ = 0.154056 nm), and Fourier transform infrared (FTIR: NICOLET IS-50, Lahore, Pakistan) spectrum to confirm the structure of the NPs. Morphology of the NPs were investigated by transmission electron microscopy (TEM: JEOL 2011, Beijing, China) and energy dispersive X-ray spectroscopy (EDS) integrated with a field emission scanning electron microscope (FE-SEM: HITACHI S-4800, Beijing, China. Temperature-dependent magnetic measurements were done by a physical property measurement system (PPMS: Quantum Design, 9T, Beijing, China) with a maximum applied field of ±30 kOe.

## 3. Results and Discussion

### 3.1. Structure and Phase Analysis

**Figure 1:**
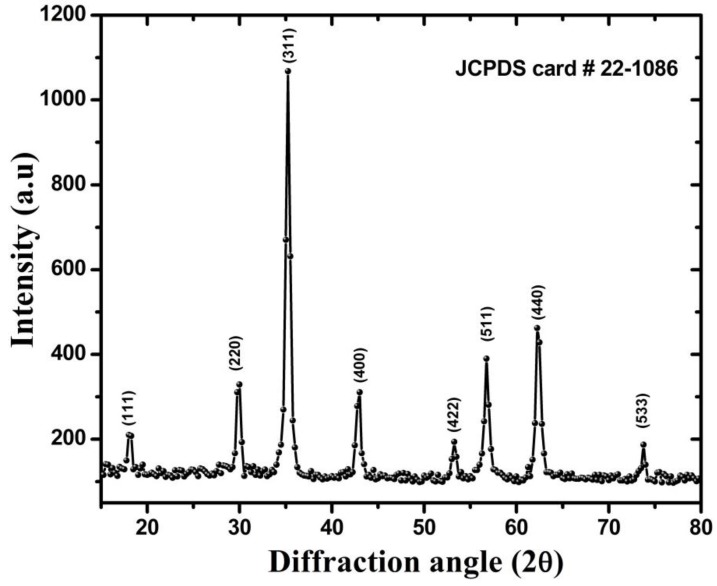
X-ray diffraction (XRD) pattern for spinel Co_0.8_Mn_0.2_Fe_2_O_4_ (CMF) nanoparticles (NPs).

XRD pattern of the powder sample for CMF is represented in Figure 1. Structural parameters including crystallite size, lattice constant, and X-ray density were calculated from the XRD pattern. The pattern shows diffraction peaks at 2θ = 18°, 30°, 35°, 43°, 53°, 57°, 63°, and 74° which correspond to (111), (220), (311), (400), (422), (511), and (440) diffraction planes. The obtained Bragg’s peaks are well matched with the standard JCPDS card No. 22-1086 for CoFe_2_O_4_. Figure 1 confirms the formation of a single-phase inverse spinel structure without any impurity peak. Crystallite size of NPs was determined from the strongest peak of XRD using Scherrer’s formula [21]:

D = 0.9λ/βcosθ
(1)

where, λ is the wavelength of radiation used, β and θ is full width at half maximum (FWHM) and angle of strongest intensity peak, respectively. In the XRD pattern (311) was the strongest peak and the calculated crystallite size was found to be 14.33 nm.

Lattice constant “*a*” can be calculated from miller indices *(h k l)* using the relation [21]:

(2) $$a=d_{hkl}\sqrt{h^{2}+k^{2}+l^{2}}$$

where *d* is the interplanar distance which is calculated from Bragg’s law. The value of lattice constant for CMF NPs is 8.439 Å. The shortest distance between magnetic ions occupied at tetrahedral (A) and octahedral (B) lattice sites considering lattice constant, known as hopping length, can be calculated by using following relation:

(3) $$L_{A}=\frac{a\sqrt{3}}{4}\;\text{and}\;L_{B}=\frac{a\sqrt{2}}{4}$$

Tetrahedral and octahedral bond lengths can also be calculated by using *a* and oxygen positional parameter u (0.381 Å) values using the following equations [23]:

(4) dAx=a3(u−14)

(5) $$d_{\mathrm{Bx}}=a{\left[3u^{2}-(11/4)u+(43/64)\right]}^{1/2}$$

where *d*_Ax_ and *d*_Bx_ represent tetrahedral and octahedral bond lengths, respectively. The calculated hopping and bond length values for tetrahedral (A) and octahedral (B) lattice sites are tabulated in Table 1.

Theoretical X-ray density can be estimated from XRD pattern using the relation [24]:

(6) $$d_{x}=\sum^{\text{​}}\frac{A}{N\times V}$$

where *A* is the sum of atomic weights of all atoms in the unit cell, *N* is Avogadro’s number, and *V* is the volume of the unit cell. In spinel structure each primitive cell consists of eight molecules, so in our case the above relation can be rewritten as:

(7) $$d_{x}=\frac{8M}{N\times a^{3}}$$

where *M* is the molecular weight of the nanoparticle and *a*^3^ is volume of cubic unit cell. The calculated value of X-ray density of CMF NPs was found to be 5.167 g/cm^3^ as given in Table 1.

### 3.2. TEM Analysis

**Figure 2:**
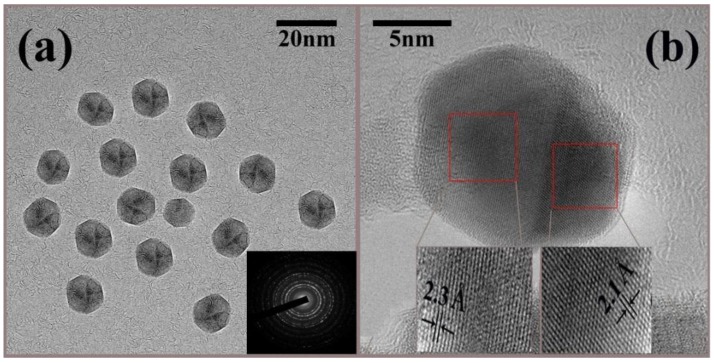
Transmission electron microscopy (TEM) images showing (**a**) spherical CMF NPs, whereas inset corresponds to selected area electron diffraction (SAED) pattern of NPs and (**b**) high resolution TEM (HRTEM) of single nanoparticle and insets belong to inverse fast Fourier transformation (IFFT) with interplanar distances of two regions of the twin boundary.

TEM images of CMF sample are presented in Figure 2. The micrograph shown in Figure 2a is taken at relatively low magnification in which it is seen that most of the NPs are nearly monodispersed and of spherical shapes with uniform distribution in diameter. The average particle size comes to be 14 nm, which is in good agreement with size inferred from XRD analysis. Furthermore, the selected area electron diffraction (SAED) pattern indicating the polycrystalline nature of the NPs (inset of Figure 2a). The bright rings correspond to different diffraction planes in the single unit cell. Figure 2b shows high resolution TEM (HRTEM) image indicating atomic planes with different orientations in a single nanoparticle. In this case it is seen that every NP has twin boundaries with different atomic planes. To investigate the morphology in detail inverse fast Fourier transformations (IFFTs) have been employed across two different regions of NP as shown in the inset of Figure 2b. The left and right red squares in Figure 2b corresponds to the cubic spinel structure of ferrite. In addition, IFFT corresponds to approximate lattice spacing as shown at the bottom Figure 2b e.g., 2.3 Å (left square) and 2.1 Å (right square).

**Figure 3:**
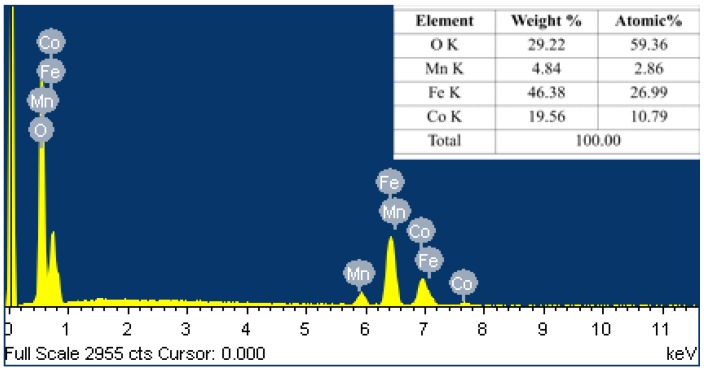
Energy dispersive X-ray spectroscopy (EDS) spectrum of spinel CMF NPs.

The EDS spectrum of the NPs is shown in Figure 3, which gives the quantitative and qualitative analyses of chemical composition of the NPs. The spectrum shows the existence of Co, Mn, Fe, and O in the sample. The EDS graph clearly indicates that the NPs did not contain any impurity elements. The inset of Figure 3 shows the elemental composition of synthesized CMF NPs and it can be seen that Co and Mn are present in 19.56 and 4.84 weight percent. This gives the presence of these metal cations by 80% and 20% in accordance with the initial stoichiometric ratio.

### 3.3. FTIR Spectroscopy

**Figure 4:**
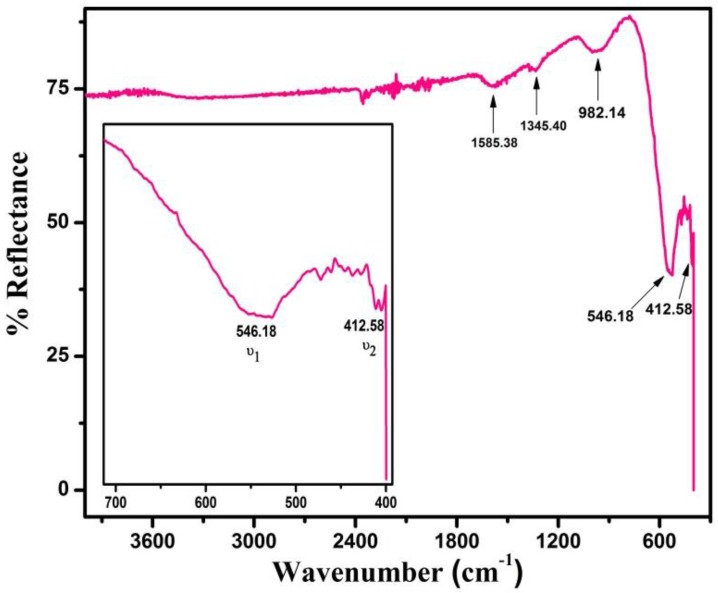
FTIR spectrum of CMF NPs taken in the range from 400 to 4000 cm^−1^.

Fourier transform infrared (FTIR) spectroscopy was used to investigate the structure and cation distribution between tetrahedral and octahedral lattice sites in inverse spinel ferrite [25]. Figure 4 shows the infrared spectrum of Mn-substituted CoFe_2_O_4_ NPs taken at room temperature in ATR mode. Generally, for spinel ferrite structures, two strong absorption bands (ʋ_1_, ʋ_2_) appear in the range of 400–600 cm^−1^ [26]. According to spinel structure of ferrites, metal ions are distributed between two sub-lattices (tetrahedral and octahedral sites) with oxygen as the nearest neighbor. The higher band (ʋ_1_) corresponds to intrinsic stretching vibrations of metal (M–O) at tetrahedral lattice sites, whereas the lower band (ʋ_2_) represents stretching vibrations of metal ions at octahedral lattice sites [27]. In our sample the higher band (ʋ_1_) appears at 546.18 cm^−1^ while the lower band (ʋ_2_) appears at 412.58 cm^−1^, as shown in Figure 4. These absorption bands reveal the formation of the cubic spinel structure, which is in agreement with XRD results of the samples.

The difference in vibrational frequency of the higher (ʋ_1_) and lower (ʋ_2_) band is attributed to the presence of more covalent bonding of Fe^3+^–O^2−^ ions at tetrahedral (A) sites as compared to octahedral lattice sites. Furthermore, the splitting of absorption band is considered due to presence of different metal cations on octahedral (B) lattice sites, like Co^2+^, Mn^2+^, Fe^2+^, and Fe^3+^ [28]. In order to determine the strength of bonding at higher and lower vibrational frequencies we can find the force constant at two (A and B) lattice sites. The force constant on these sites can be calculated by using the following relation [29]:

(8) $$F=4\pi c^{2}{ʋ}^{2}m$$

where *c* is speed of light, ʋ is vibrational frequency of cations at tetrahedral and octahedral sites, and *m* is reduced mass of Fe^3+^ and O^2−^ ions. Based on this equation, the calculated values of vibrational frequencies and force constants at A and B sites are listed in Table 1.

In Figure 4 the peaks obtained in FTIR at 982 cm^−1^ and 1345 cm^−1^ are assigned to C–H bending and C–O–C symmetrical stretching vibrations, while the peak observed at 1585 cm^−1^ originates from C=O stretching vibrations in the spinel structure [30,31]. The appearance of bands around 2100–2370 cm^−1^ are due to the atmospheric CO_2_ which is absorbed on the surface of NPs during the FTIR measurements [6].

### 3.4. Magnetic Analysis

**Figure 5:**
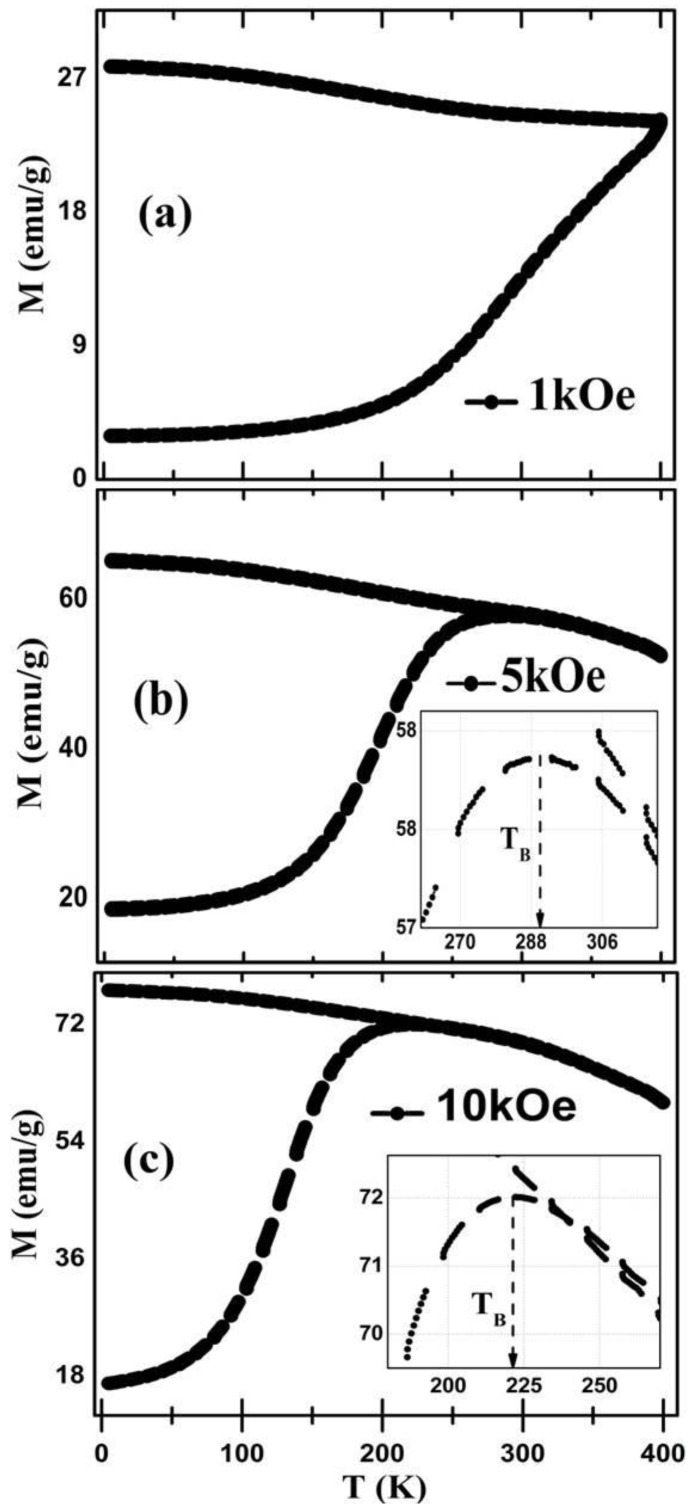
Zero field cooled-field cooled (ZFC-FC) curves of CMF at *H* = (**a**) 1kOe; (**b**) 5kOe; and (**c**) 10kOe.

To get information about magnetic properties of CMF NPs, zero field cooled (ZFC) and field cooled (FC) magnetization curves were recorded in the temperature range of 5–400 K with an applied field varying from 1 kOe to 10 kOe. ZFC and FC magnetization curves under the applied field of 1 kOe, 5 kOe, and 10 kOe are represented in Figure 5a–c, respectively.

The irreversibility in ZFC and FC curves occurred at 288 K and 224 K at 5 kOe and 10 kOe, respectively, and above the mentioned temperature, the NPs showed a super-paramagnetic state as shown in Figure 5b,c. It is reported in literature that flattening in FC curves after bifurcation represents interparticle coupling, whereas an increase in the FC curve depicts non-interactions between the particles [32]. In the present case it is observed that magnetization in the FC curve increases monotonically at temperature ≤116 K, 80 K, and 50 K at 1 kOe, 5 kOe, and 10 kOe, respectively, which corresponds to non-interacting regions. Below these regions, flattening in FC curves corresponds to interactions. These interactions might be attributed to the AFM dopant (Mn) with cobalt ferrite, since the Neel temperature of Mn is 116 K [33].

In Figure 5b,c ZFC magnetization exhibits a sharp cusp at 5 kOe and 10 kOe applied field at 290 ± 5 K and 220 ± 5 K, respectively. This peak point is known as the blocking temperature (*T*_b_); after this sharp peak magnetization tends to fall rapidly. The sudden decrease in the magnetization of ZFC after *T*_b_ is attributed to spin glass behavior of strongly-interacting particles in a magnetic system [34]. However, in the case of the ZFC-FC curve at 1kOe no sharp cusp and fine coincidence/irreversibility point has been observed up to 400 K. This observation helps to understand the dependence of bifurcation and blocking temperature on the applied field. This suggests that with an increase in cooling field the ZFC-FC cycles becomes broader, and *T*_b_ and *T*_irr_ shift towards lower temperatures. This behavior typically identifies the super-paramagnetism below *T*_irr_ and strong dipolar interactions among the particles [35].

**Figure 6:**
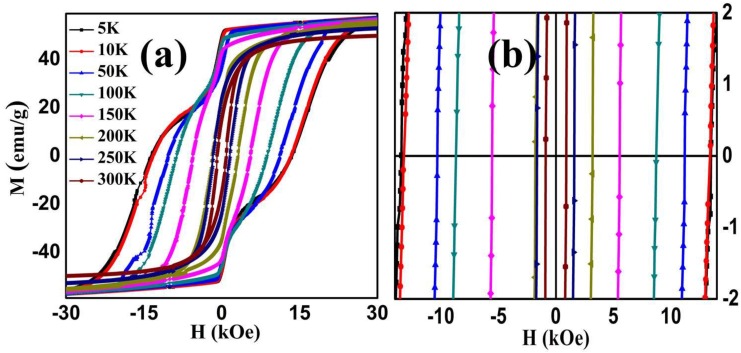
(**a**) Magnetic hysteresis loops of CMF NPs showing ferromagnetic and wasp-waist shape, taken at various temperatures ranging from 5 to 300 K and (**b**) extended view of coercivity near the origin.

Figure 6 represents magnetic hysteresis (M–H) loops of synthesized NPs under a FC state with an applied field of 1 kOe. The inset of the figure shows detailed hysteresis loops near the origin, at different temperatures, to make coercivity visible. It can be seen from Figure 6 that hysteresis loops show “*kink or wasp-waisted*” behavior at temperatures below 150 K, while above 150 K this “*wasp-waist*” effect is negligible. Various reasons have been reported for such type of loops including magnetic coupling between the two different magnetic phases (*i.e.*, hard Co and soft Mn) with different coercivities [36] or reordering of magnetic spins below 150 K under the influence of the applied field. These spin reorientations are responsible for constrained on M–H loop, which can be explained by considering the domain wall motion and pinning of the potential wells formed by the directional order [37]. This resultantly alters the magnetic properties of the samples. Another reason may be the oxidation of soft magnetic layers in the presence of atmospheric oxygen [38]. This type of behavior arises due to a mixture of grain boundaries and combination of magnetic composites possessing different magnetic properties [39]. In our case we believe that “*wasp-waist*” behavior in M–H loops arises as a result of coexistence of 80% Co (hard) and 20% Mn (soft) phases in the samples.

**Figure 7:**
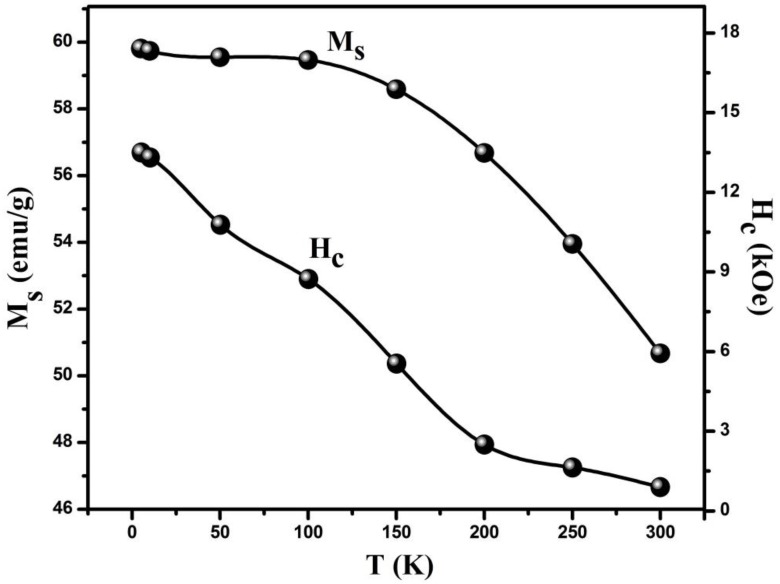
Effect of temperature on coercivity (right side) and saturation magnetization (left side) of CMF NPs.

It is observed that M–H loops presents a noteworthy increase in coercivity and saturation magnetization of the sample as the temperature goes down to 5 K. The calculated values of coercivity from M–H loops at different temperatures are shown in Figure 7 (right side). This increasing behavior of coercivity can be understood by considering that at low temperature magnetic anisotropy increases and particles scatter in the direction of the anisotropic field due to which coercivity increases. In the NPs, the effect of thermal fluctuations of blocked moments across the anisotropy barrier is responsible for the enhancement in coercivity [40] at low temperatures. Therefore, with decreasing temperature, the reduced thermal fluctuations tend to make magnetic moments isotropic, causing an increase in coercivity of the system [41].

The saturation magnetization (*M*_s_) of CMF calculated from M–H loops is shown in Figure 7 (left side). At room temperature, magnetization mainly depends on size effects, whereas at low temperature size confinement and the quantum effects, spin glass transitions, and thermal dependence might be considered as the possible reason for the increase in magnetization of NPs [42]. Apart from this, at the nanoscale, it is assumed that a nanoparticle is quantized of spin wave excitation. At high temperature, particles possess broader energy levels with a continuous excitation spectrum. In such a case the temperature dependence of magnetization can be ascribed, like in bulk materials, followed by Bloch’s law [43]. While at low temperatures it is considered that at finite size nanoparticles’ long spin waves’ excitations cannot propagate, so the spectrum becomes discrete, which can be attributed to the increase in magnetization at low temperature. This behavior can be explained from following equation [44]:

(9) $$M_{s}\left(T\right)=\;M_{s}\left(0\right)-C\left[e^{\left(-\frac{E_{1}}{K_{B}T}\right)}+\;e^{\left(-\frac{E_{2}}{K_{B}T}\right)}\right]$$

where *M*_s_ (*T*) is temperature-dependent magnetization, *M*_s_ (0) is magnetization at 0 K, C is constant (C= *M*_s_(0)/*N*n), depending on number of modes (*N*), and occupancy state (n), *E*_1_ and *E*_2_ denotes energy levels, and *K*_B_ is the Boltzman constant. This shift in the spin wave spectrum relative to the temperature can alter the population in magnetic energy levels and, resultantly, magnetic response increases as indicated by the FC curve.

Magnetic moment per formula unit (*n*_B_) of CMF NPs at different temperatures can be calculated by using following relation [45]:

(10) $$n_{B}=\frac{M\times M_{s}}{5585}$$

where *M* is the molecular weight of the nanoparticle, *M*_s_ is the saturation magnetization measured at different temperatures. As in ferrites, magnetization strongly depends on cationic distribution between tetrahedral (A) and octahedral (B) sites and the spins in these sites are oppositely aligned, leading these materials to act ferromagnetically in nature. Similarly, the effective anisotropy constant (*K*) of the NPs can be calculated at different temperatures using the relation [46]:

(11) $$H_{c}=\frac{K_{eff}\times 0.96}{M_{s}}$$

where *H*_c_ is the coercivity and *M*_s_ is the saturation magnetization at a particular temperature. According to the above relation, the anisotropy constant has direct relation with the coercivity of the sample. Therefore, with decreasing temperature the effective anisotropy constant, as well as the coercivity of the system, increases. The strong anisotropy of the synthesized sample primarily depends on the presence of Co^2+^ ions on octahedral (B) sites in the spinel structure [47]. The calculated values of magnetic moment and anisotropy constant at different temperatures are listed in Table 2.

From Table 2 it is seen that all magnetic parameters (*i.e.*, coercivity, saturation magnetization, magnetic moment, and anisotropy constant) show an increasing trend as the temperature of the system decreases. This is due to the fact that the anisotropy energy is dominating the thermal energy as the temperature of the system drops.

## 4. Conclusions

In this paper the structural and magnetic properties of CMF NPs annealed at 600 °C via a co-precipitation route were investigated. The purity of NPs and crystalline nature was confirmed by XRD measurements. TEM images show ultrafine NPs with uniform morphology. FTIR spectrum shows two absorption bands around 546.18 cm^−1^ and 412.58 cm^−1^ which represent intrinsic metal oxide stretching vibrations at tetrahedral and octahedral lattice sites. Magnetic properties were explored as a function of temperature, ranging from 5 to 400 K. The magnetization *versus* temperature plots under ZFC and FC modes show the shift in blocking temperature with an increase in the applied field. The high magnetic properties of synthesized NPs suggest that the obtained nanocrystalline magnetic ferrites can be used for practical applications in spintronics.

## Figures and Tables

**Table 1:** Structural parameters of 20% Mn substituted CoFe_2_O_4_ nanoparticles calculated from XRD and Fourier transform infrared (FTIR) spectrum.

Parameters	Values
Crystallite Size	14.33 nm
Lattice parameter	8.439 Å
Tetrahedral hopping length (*L*_A_)	3.654 Å
Octahedral hopping length (*L*_B_)	2.983 Å
Tetrahedral bond length (*d*_Ax_)	1.914 Å
Octahedral bond length (*d*_Ax_)	2.060 Å
X-ray density	5.167 g/cm^3^
Higher vibrational frequency band (ʋ_1_)	546.18 cm^−1^
Lower vibrational frequency band (ʋ_2_)	412.58 cm^−1^
Force constant at A site	2.16 × 10^5^ dyne/cm^2^
Force constant at B site	1.23 × 10^5^ dyne/cm^2^

**Table 2:** Temperature-dependent magnetic parameters of Mn*_x_*Co_1-*x*_Fe_2_O_4_ (*x* = 0.2) nanoparticles calculated from M–H loops taken from the physical property measurement system.

Temperature (K)	*H*c (Oe)	*M*s (emu/g)	*n*_B_ (×10^−2^)	*K* × 10^3^ (erg/Oe)
5	13,499.75	59.81	2.503	841.06
10	13,309.22	59.74	2.500	828.22
50	10,777.68	59.55	2.492	668.55
100	8724.59	59.47	2.489	540.47
150	5542.09	58.59	2.452	338.24
200	2490.22	56.68	2.372	147.02
250	1625.93	53.95	2.258	91.26
300	893.41	50.67	2.120	47.15
